# Rho Kinase Activity, Connexin 40, and Atrial Fibrillation: Mechanistic Insights from End-Stage Renal Disease on Dialysis Patients

**DOI:** 10.3390/jcm9010165

**Published:** 2020-01-07

**Authors:** Lorenzo A. Calò, Verdiana Ravarotto, Giovanni Bertoldi, Elisa Pagnin, Barbara Rossi, Matteo Rigato, Paul A. Davis, Riccardo Proietti

**Affiliations:** 1 Department of Medicine, Nephrology, Dialysis and Transplantation Unit, University of Padova, 35128 Padova, Italy; verdiana.ravarotto@gmail.com (V.R.); giovanni.bertoldi92@gmail.com (G.B.); elisa.pagnin@unipd.it (E.P.); barbara.rossi@aopd.veneto.it (B.R.); matteo.rigato@hotmail.it (M.R.); 2 Department of Nutrition, University of California, Davis, CA 95616, USA; padavis@ucdavis.edu; 3 Department of Cardiac, Thoracic, Vascular Sciences and Public Health, University of Padova, 35128 Padova, Italy; riccardoproietti6@gmail.com

**Keywords:** atrial fibrillation, Rho kinase, connexin, cardiovascular–renal remodeling, renal failure

## Abstract

Evidence on cellular/molecular mechanisms leading to atrial fibrillation (AF) are scanty. Increased expression of Rho kinase (ROCK) and myosin-phosphatase-target subunit-1 (MYPT-1), ROCK activity’s marker, were shown in AF patients, which correlated with connexin 40 (Cx40) expression, membrane protein of heart gap junctions, key for rapid action potential’s cell–cell transfer. AF is the most frequent arrhythmia in dialysis patients who present increased MYPT-1 phosphorylation, which correlates with left ventricular (LV) mass. Given ROCK’s established role in cardiovascular–renal remodeling, induction of impaired cell-to-cell coupling/potential conduction promoting AF initiation/perpetuation, we evaluated in dialysis patients with AF, MYPT-1 phosphorylation, Cx40 expression, and their relationships to support their involvement in AF. Mononuclear cells’ MYPT-1 phosphorylation, Cx40 expression, and the ROCK inhibitor fasudil’s effect were assessed in dialysis patients with AF (DPAFs), dialysis patients with sinus rhythm (DPs), and healthy subjects (C) (western blot). M-mode echocardiography assessed LV mass and left atrial systolic volume. DPAF’s phospho-MYPT-1 was increased vs. that of DPs and C (1.57 ± 0.17 d.u. vs. 0.69 ± 0.04 vs. 0.51 ± 0.05 respectively, *p* < 0.0001). DP’s phospho-MYPT-1 was higher vs. that of C, *p* = 0.009. DPAF’s Cx40 was higher vs. that of DPs and C (1.23 ± 0.12 vs. 0.74 ± 0.03 vs. 0.69 ± 0.03, *p* < 0.0001). DPAF’s phospho-MYPT-1 correlated with Cx40 (*p* < 0.001), left atrial systolic volume (*p* = 0.013), and LV mass (*p* = 0.014). In DPAFs, fasudil reduced MYPT-1 phosphorylation (*p* < 0.01) and Cx40 expression (*p* = 0.03). These data point toward ROCK and Cx40’s role in the mechanism(s) leading to AF in dialysis patients. Exploration of the ROCK pathway in AF could contribute to AF generation’s mechanistic explanations and likely identify potential pharmacologic targets for translation into treatment.

## 1. Introduction

Atrial fibrillation (AF) is a clinically common sustained arrhythmia [1], where atrial myocytes structural and electrical remodeling and cardiac remodeling contribute greatly to its pathogenesis [2].

The renin–angiotensin–aldosterone system (RAAS) has been implicated as a major signal-processing pathway in mediating the molecular mechanisms involved in the development of atrial fibrosis and AF [3]. The identification of a central role played by RAAS in the cellular events leading to AF derives not only from clinical trials documenting the efficacy of RAAS blockade for primary and secondary prevention of AF but also by its providing an explanation for the epidemiological associations consistently noted for AF, hypertension, and renal disease [4]. While the RAAS system is very complex as it encompasses an ever increasing number of cellular signal transduction pathways, identification and exploration of RAAS system pathways associated with increased atrial fibrosis and cellular electrical disturbances is likely to be important not only on the clinical ground but may also be of direct relevance to the development of new pharmacological therapies.

The links between RAAS and fibrosis necessarily involve the main RAAS effector hormone, angiotensin II (Ang II), which binds and activates the type 1 Ang II receptor (AT1R). Reactive oxygen species (ROS) are major downstream mediators of AT1R activation and are responsible for the activation of mitogen-activated protein kinase (MAPK), which is directly implicated in fibrosis and remodeling. Indeed, AT1R as well as TGFβ1 are direct activators of NADPH oxidase [3]. An alternative pathway coupled to AT1R activation is the activation of PKC (protein kinase C) and the consequent upregulation of nuclear factor-kB and activator protein [1] in response to a phospholipase C-mediated increase of phosphatidyl inositol. Most recently, Janus kinase (JAK) and its downstream effectors have been shown to be regulated by AT1R activation and to be involved in atrial fibrosis and remodeling of failing hearts [5].

Connexins (Cxs) are integral membrane proteins of heart cell gap junctions, fundamental for the rapid cell–cell transfer of action potential [6,7]. While changes in Cxs are recognized as hallmarks of atrial structural remodeling, their specific interactions with Ang II are less well documented. In an animal model of chronic kidney disease, Qiu et al. [8] showed that an increased susceptibility to AF induction assessed electrophysiologically was linked to associated cardiac remodeling via reduced expression of Cx40 and an increase of both Cx43 and N-cadherin. Moreover, the authors reported that the Cxs changes found were mediated by Ang II through AT1R activation of the protein of the Rho GTPase superfamily [8]. This is noteworthy as the RhoA/Rho kinase (ROCK) pathway plays a pivotal role in the development and maintenance of hypertension in animal models and in humans [9]. This last finding then raised questions as to the exact role(s) of the RhoA/ROCK pathway of Ang II transduction in the molecular mechanisms linked to AF. Just recently, Chen et al. [10] using human left atrial appendages from patients undergoing cardiac surgery found an overexpression of ROCK, its metabolic downstream mediator myosin phosphatase target subunit-1 (MYPT-1), known as the marker of ROCK activity, as well as Cx40 in those patients with documented AF.

The current study sought to quantify in circulating mononuclear cells from a small cohort of patients with end-stage renal failure on dialysis the effect of having either sinus rhythm or AF on MYPT-1 phosphorylation as a marker for RhoA/ROCK pathway activation as well as Cx40 level and to evaluate their relationship. The study also compared mononuclear cells’ MYPT-1 phosphorylation status and Cx40 level found in both AF and sinus rhythm dialysis patients to those found in a group of healthy individuals used as the control group and evaluated the effect of fasudil, a ROCK inhibitor, on MYPT-1 phosphorylation and Cx40 expression.

## 2. Patients and Methods

### 2.1. Patients

Subjects with end-stage renal disease under chronic dialysis were selected from the cohort of patients of the Nephrology, Dialysis, and Transplantation Unit at the Department of Medicine, University of Padova. Twenty-two subjects were recruited, with half of them having been diagnosed with permanent AF (*n* = 11) (DPAF), seven males, four females, age range 49–81; the other half had no AF (*n* = 11) (DP), seven males, four females, age range 53–82. Both DPAF and DP dialysis patients were under chronic dialysis with low-flux bicarbonate dialysis with ultrapure dialysate, using a polysulfone dialyzer 1.8 m^2^, 210–240 min three times a week, for at least one year (range 1–5 years). All participating dialysis patients had the vascular access via the arteriovenous fistula and a mean Kt/V ratio of 1.43 ± 0.07.

The following criteria were used for patients’ selection: the lack of co-morbidity such as chronic obstructive pulmonary diseases, heart failure, cancer, and lack of hospitalization in the last six months. The etiology of ESRD (end-stage renal disease) for dialysis patients was: chronic glomerulonephritis (three patients); diabetic nephropathy (eight patients); nephroangiosclerosis (four patients); adult polycystic kidney disease (one patient); IgA nephropathy (two patients); reflux nephropathy (one patient); amyloidosis (one patient); undiagnosed (two patients). All patients were also checked for the absence of C reactive protein changes chosen as a biochemical marker of inflammation (CRP 2.30 ± 1.30 mg/L) and for no clinical evidence of infectious or inflammatory disease.

All dialysis patients were under epoetin (EPO) treatment at a dose ranging from 4000 to 12,000 UI/week. Of the 11 DPAFs, six were anticoagulated with warfarin and five with low-molecular-weight heparin.

Patients’ blood pressure ranged from 135/86 to 155/92 mmHg and antihypertensive treatment included dihydropyridine calcium channel blockers such as amlodipine or lercanidipine at the full dose of 10 or 20 mg/day respectively, ACE inhibitors such as ramipril at the dose of 5 mg/day, Angiotensin II type 1 receptor blockers (ARBs) such as olmesartan at the dose of 20 mg/day and α blockers such as doxazosin at the dose of 2 or 4 mg/day, and different combinations of these drugs. None of the patients was under lipid-lowering treatment.

Vitamin D (1.25 dihydroxyvitamin D3, 25 mg every two days) and calcium supplements were present in the therapeutic regimen in 10 patients. A majority of the patients were treated with PO_4_ binders, with two patients treated with sevelamer HCl (3200–4000 mg/day), seven treated with calcium carbonate (2500–3000 mg/day), and three treated with lanthanum carbonate (2250 mg/day).

All dialysis patients were treated with supplements of folic acid (10 mg) after the dialysis session.

A control group of healthy subjects (*n* = 11), seven males, four females, age range 37–65 were recruited from the staff of the University of Padova Nephrology, Dialysis, and Transplantation Unit.

The investigation conforms with the principles outlined in the Declaration of Helsinki. Informed consent was obtained from all study participants.

### 2.2. Methods

#### 2.2.1. Mononuclear Cell Preparation

Blood samples from patients and controls were processed the same day immediately after the collection and peripheral blood mononuclear cells (PBMCs) were obtained after plasma separation from 35 mL of EDTA anticoagulated blood and were isolated by density gradient with Lympholyte-H (Cedarlane, Burlington, ON, Canada).

#### 2.2.2. MYPT-1 and Cx40 Western Blot Analysis

Protein expression and MYPT-1 phosphorylation state were assessed by western blot analysis as previously reported [9]. Briefly, total PBMC protein extract from patients and controls was obtained by cell lysis with a buffer solution (Tris HCl 20 mmol/L, NaCl 150 mmol/L, EDTA 5.0 mmol/L, Niaproof 1.5%, NaVO_4_ 1.0 mmol/L, SDS 0.1%, PMSF 0.5 mmol/L) supplemented by Protease Inhibitor Cocktail (Roche Molecular Biochemicals, Mannheim, Germany) and Phosphatase Inhibitor Cocktail I (Sigma-Aldrich, St. Louis, MO, USA). All samples were placed on ice and underwent three cycles of sonication using a UP200S sonicator (Hielscher GmbH, Teltow, Germany). The supernatant was recovered and stored at −80 °C for the subsequent analysis. After protein quantification with bicinchoninic assay (BCA Protein Assay, Pierce, Rockford, IL, USA), samples were separated by SDS-PAGE in Tris pH 8.3 and subsequently transferred onto nitrocellulose membranes using a Hoefer TE 22 Mini Tank Transfer Unit (Amersham Pharmacia Biotech, Uppsala, Sweden) using a 39 mmol/L glycine, 48 mmol/L Tris base, 0.037% SDS (electrophoresis grade) and 20% methanol transfer buffer. Membranes were then blocked with a non-fat milk solution (5% in Tween-PBS) and subsequently probed with primary polyclonal anti-phospho-MYPT-1 (Thr853) or anti-MYPT-1 antibodies (Cell Signaling Danvers, MA, USA) and anti-Cx40 (Abcam, Cambridge, UK) overnight. To normalize the results, antimonoclonal antibodies were used to detect β-actin (Sigma-Aldrich, St. Louis, MO, USA) levels in each membrane. Specific HRP-conjugated secondary antibodies were used (Amersham Pharmacia, Uppsala, Sweden) and immunocomplexes were visualized by chemiluminescence using SuperSignal West Pico Chemiluminescent Substrate (Pierce) in an Amersham Imager 600 (GE Healthcare UK Limited, Buckinghamshire, UK).

Protein immunocomplex levels were evaluated by a densitometric semi-quantitative analysis of the gray-scale 16-bit tiff output file using NIH ImageJ software (NIH, Bethesda, MD, USA). The quantification of the targeted proteins was normalized using the quantification of the housekeeping protein observed for the same membrane. Finally, the ratio between phospho-MYPT-1 and β-actin to MYPT-1 and β-actin was used as an index of ROCK activity.

#### 2.2.3. Effect of ROCK Inhibition with Fasudil on MYPT-1 Phosphorylation and Cx40 Expression

To assess the relationship between ROCK activity and Cx40 expression, ROCK activity was blocked using the ROCK inhibitor fasudil (Sigma Aldrich, St. Louis, MO, USA). Briefly, three aliquots of 5 × 10^6^ PBMCs from five representative dialysis patients with atrial fibrillation, were incubated in medium RPMI-1640 (Sigma Aldrich, St. Louis, MO, USA) with zero, 500 and 1000 µM fasudil for 3 hours at 37 °C. Cells were then washed three times with PBS and total protein extracts obtained and analyzed as detailed above, quantifying MYPT-1 phosphorylation and Cx40 expression.

#### 2.2.4. Echocardiography

M-mode echocardiography according to Devereux and coworkers [11] was used to measure LV mass normalized for body surface area. Normal values were defined according to guidelines: <116 g/m^2^ for males and <96 g/m^2^ for females [12]. Relative wall thickness was calculated according to guidelines and left ventricular hypertrophy (LVH) was considered as concentric if ≥0.42 and eccentric if <0.42 [13]. Left atrial systolic volume was also measured.

#### 2.2.5. Statistical Analysis

Continuous variables were tested for normality using the Kolmogorov–Smirnov test and expressed as mean ± standard deviation for normally distributed data or median (lower quartile − upper quartile) for non-normally distributed data. Error bars represent standard error of the mean (SEM). Categorical variables were expressed as frequency (percentage). Group differences were tested using ANOVA as appropriate. *p* value <0.05 defined statistical significance. The Pearson product–moment correlation coefficient, r, was used to measure associations between variables. Statistical analysis was performed using SPSS Version 22 (IBM, New York, NY, USA).

## 3. Results

### 3.1. MYPT-1 Phosphorylation Status

In patients with end-stage renal failure on dialysis, the ratio of phosphorylated MYPT-1 to MYPT-1 in PBMC increased in dialysis patients with AF (DPAFs) compared to patients on dialysis without AF (DPs) as well as versus healthy controls (C): 1.57 ± 0.17 vs. 0.69 ± 0.04 vs. 0.51 ± 0.05 respectively, ANOVA: *p* < 0.0001.

**Figure 1:**
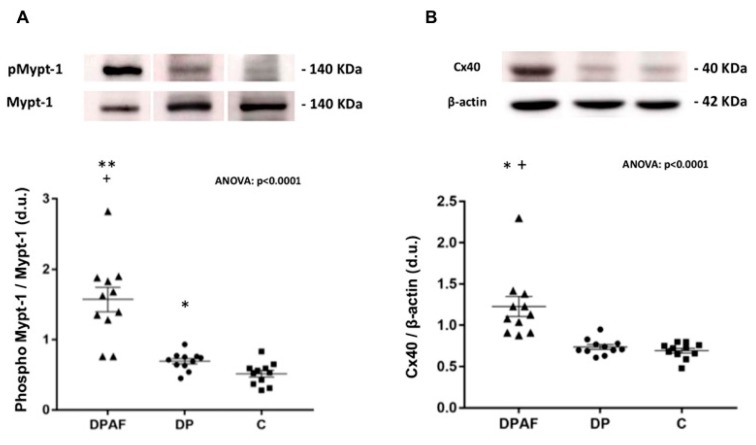
(**A**) Densitometric analysis of phospho-MYPT-1 (myosin-phosphatase-target subunit-1) to MYPT-1 ratio in mononuclear cells of dialyzed patients with atrial fibrillation (DPAFs) (*n* = 11), dialyzed patients without AF (DPs) (*n* = 11), and healthy subjects (C) (*n* = 11). The top of the figure shows representative phosphorylated phospho-MYPT-1 and MYPT-1 western blot products from one DPAF, one DP, and one healthy control subject. **: *p* < 0.0001 vs. DP; +: *p* < 0.0001 vs. C; *: *p* = 0.009 vs. C. (**B**) Densitometric analysis of connexin 40 (Cx40) to β-actin ratio in mononuclear cells of dialyzed patients with AF (DPAFs) (*n* = 11), dialyzed patients without AF (DPs) (*n* = 11), and healthy subjects (C) (*n* = 11). The top of the figure shows representative Cx40 and β-actin western blot products from one DPAF, one DP, and one healthy control subject. *: *p* < 0.001 vs. DP; +: *p* < 0.001 vs. C.

In particular, the level of phospho-MYPT-1 in DPAFs was significantly higher compared to that in both DP and C (*p* < 0.0001 vs. both DP and C). The level of phospho-MYPT-1 was also significantly higher in DP compared to that in C, (*p* = 0.009) (Figure 1A).

### 3.2. Cx40 Protein Level

The mononuclear cell level of Cx40 was significantly higher in DPAFs compared to that in DPs and C groups: 1.23 ± 0.12 vs. 0.74 ± 0.03 vs. 0.69 ± 0.03, respectively, ANOVA: *p* < 0.0001 (Figure 1B). In particular, the level of Cx40 in DPAFs was significantly higher compared to that of both DPs and C (*p* < 0.001 vs. both DPs and C). Although the level of Cx40 appeared higher in DPs than in C, the difference did not reach statistical significance.

### 3.3. Correlation Analysis between MYPT-1 Phosphorylation and Cx40 Protein Level, MYPT-1 Phosphorylation and Left Atrial Systolic Volume, MYPT-1 Phosphorylation and Cardiac Mass, and Cardiac Mass and Left Atrial Systolic Volume in DPAFs

**Figure 2:**
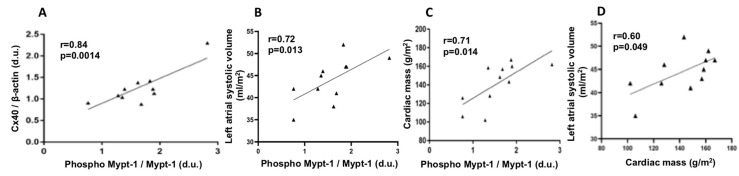
(**A**) Correlation analysis between phospho-MYPT-1 and Cx40 in DPAFs. (**B**) Correlation analysis between phospho-MYPT-1 and left atrial systolic volume. (**C**) Correlation analysis between phospho-MYPT-1 and cardiac mass. (**D**) Correlation analysis between cardiac mass and left atrial systolic volume in DPAFs.

In DPAFs, a statistically significant linear correlation (*r* = 0.84, *p* = 0.0014) was found between the MYPT-1 phosphorylation status and Cx40 protein expression levels. In addition, MYPT-1 phosphorylation state was also significantly correlated with left atrial systolic volume (*r* = 0.72, *p* = 0.013 and with cardiac mass (*r* = 071, *p* = 0.014,). In DPAFs, cardiac mass and left atrial systolic volume were also significantly correlated (*r* = 0.60, *p* = 0.049), (Figure 2).

### 3.4. Effect of ROCK Inhibition with Fasudil on MYPT-1 Phosphorylation and Cx40 Expression

**Figure 3:**
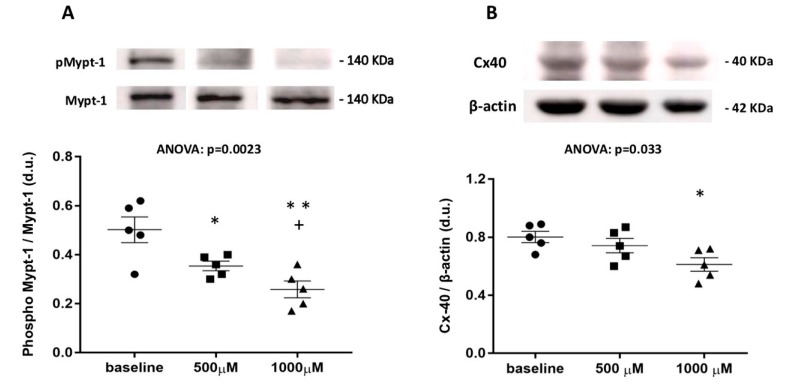
(**A**) Densitometric analysis of phospho-MYPT-1 in mononuclear cells of five DPAFs after incubation with 500 and 1000 μM of fasudil. The top of the figure shows phosphorylated phospho-MYPT-1 western blot products from one representative patient out of five. **: *p* = 0.005 vs. baseline; +: *p* = 0.04 vs. 500 μM; *: *p* = 0.03 vs. baseline. (**B**) densitometric analysis of connexin 40 in mononuclear cells of five DPAFs after incubation with 500 and 1000 μM of fasudil. The top of the figure shows connexin 40 western blot products from one representative patient out of five. *: *p* = 0.014 vs. baseline.

Incubation of PBMCs, of five representative DPAFs with two different concentrations of Fasudil (500 and 1000 μM) inhibited MYPT-1 phosphorylation (ANOVA: *p* = 0.0023) with the phosphorylated level of MYPT-1 at baseline vs. 500 μM: 0.50 ± 0.05 vs. 0.34 ± 0.02, *p* = 0.03, and baseline vs. 1000 μM: 0.50 ± 0.05 vs. 0.26 ± 0.03, *p* = 0.005. Moreover, there was a statistically significant decline (*p* = 0.04) in MYPT-1 phosphorylation between 500 μM (0.34 ± 0.02) vs. 1000 μM (0.26 ± 0.03) fasudil (Figure 3).

The incubation of PBMCs from the same DPAFs with two different concentrations of fasudil (500 and 1000 μM) reduced Cx40 expression (ANOVA: *p* = 0.033), but showed a statistically significant reduction in Cx40 expression only at the highest concentration (1000 μM) of fasudil: 0.80 ± 0.04 (at baseline) vs. 0.74 ± 0.05 (500 μM, *p* = ns) vs. 0.61 ± 0.05 (1000 μM, *p* = 0.014). No statistically significant differences were found for 0 vs. 500 μM or 500 vs. 1000 μM fasudil (Figure 3).

### 3.5. Cardiac Remodeling and MYPT-1 Phosphorylation Relationship

Cardiac left ventricular mass was higher in DPAFs: 148.6 ± 5.3 g/m^2^ for males and 123.4 ± 19.4 g/m^2^ for females compared with reference values (<116 g/m^2^ for males and <96 g/m^2^ for females [12]. Cardiac left ventricular mass was also significantly correlated to MYPT-1 phosphorylation in these patients (*r* = 0.71, *p* = 0.014, Figure 2). Left atrial systolic volume was higher in DPAFs compared with DPs: (44.45 ± 1.38 mL/m^2^ vs. 35.09 ± 2.62, *p* = 0.005) respectively and significantly correlated with MYPT-1 phosphorylation (Figure 2). In DPAFs, cardiac mass and left atrial systolic volume were also significantly correlated (Figure 2).

## 4. Discussion

AF is characterized by remodeling in structural, electric, and contractile functions of the atrial myocytes. The major molecular mechanisms driving this atrial remodeling, which are the substrates for the development of AF include the interrelated RAAS, TGFβ1 [14], extracellular signal regulated kinases (ERK) [15], and oxidative stress pathways [16].

AF is the most frequent occurring cardiac arrhythmia in Chronic Kidney Disease (CKD) and dialysis patients, which is likely linked to an abnormal activation of the RAAS. We have previously shown that the pro-fibrotic and pro-inflammatory effects of aldosterone are mediated by the activation of the ROCK [17]. These effects include a cardiovascular (CV)–renal remodeling and impairment of cell-to-cell coupling and conduction of potential, which promote AF initiation and perpetuation by reducing atrial wavelength [7,18].

The findings of our study document that patients with end-stage renal disease (ESRD) on dialysis with permanent AF show an increased MYPT-1 phosphorylation and increased Cx40 level when compared to either patients with ESRD on dialysis without AF or healthy controls. These findings reinforce the results of our previous studies in chronic renal failure patients and ESRD patients on dialysis [19]. Patients on dialysis without AF did not have increased Cx40, and thus only the AF subset of dialysis patients have increased levels of MYPT-1 phosphorylation and Cx40, which shows a positive linear correlation.

Recently, the involvement of the ROCK pathway was documented using left atrial appendage biopsies of AF patients vs. those with sinus rhythm which showed increased expression of ROCK and MYPT-1 that correlated with the expression of Cx40, an integral membrane protein of heart gap junctions, fundamental for the rapid cell–cell transfer of action potential [10].

ROCK is a downstream Rho family protein, amply expressed in all eukaryotic cells that binds to and is a major downstream effector of the small GTPase RhoA [20]. RhoA interaction with GTP results in an active state triggering a kinase cascade reaction with a broad range of biological effects [20] including the upregulation of NADPH oxidase and induction of oxidative stress and the cardiovascular effects of oxidative stress [21,22,23], which comprise changes in calcium signaling, reduction in nitric oxide bioavailability, conduction disturbances, and contractile dysfunction [24].

We have previously demonstrated that the ROCK pathway is activated in hypertensive patients in terms of increased MYPT-1 phosphorylation and p63RhoGEF protein level [9], the latter a specific mediator transducing the Ang II message from activated AT1R to RhoA/ROCK activation [25]. RhoA/ROCK activation declined after six months of treatment with the AT1R blocker olmesartan [26], suggesting that the RhoA/ROCK pathway plays a major role in the development and maintenance of hypertension [9]. MYPT-1 is the catalytic domain of the myosin light chain phosphatase, and the inhibition of MYPT-1 activity by ROCK is the key mechanism involved in cardiovascular–renal remodeling [19]. MYPT-1 is, in fact, the target of ROCK, which, via an inhibitory phosphorylation of MYPT-1 increases the activity of myosin light chain kinase leading to cardiovascular–renal remodeling [9].

Recently, increased levels of ROCK activity as assessed by MYPT-1 phosphorylation status in circulating leucocytes were found in stable systolic heart failure patients. Moreover, in these patients, ROCK activation was inversely correlated with LV systolic function [27]. In addition, recent observations suggest that ROCK inhibition decreases cardiac remodeling and LV dysfunction [28,29,30] and inhibition of ROCK signaling in rats with pressure overload hypertrophy resulted in improved LV geometry, reduced collagen deposition, and improved diastolic function [28,31].

In a prior study, we reported higher levels of phospho-MYPT-1 were present in CKD and dialysis patients with left ventricular hypertrophy and MYPT-1 phosphorylation correlated with LV mass [19]. This has been also seen in the current study, thereby further strengthening the association of ROCK activity and cardiac remodeling in terms of LV hypertrophy in these types of patients.

The nature of the linkage of the increased MYPT-1 phosphorylation and the increased expression of Cx40 that was detected in our study in DPAFs remains to be fully defined. However, the observation that the activation of the RhoA/ROCK pathway in the dialysis patients with AF of our study is associated with an increased expression of Cx40, integral membrane protein of heart gap junctions, fundamental for the rapid cell–cell transfer of action potential, and that the increased MYPT-1 phosphorylation, the marker of ROCK activity, correlates with Cx40 in these patients adds more evidence for the involvement of the RhoA/ROCK pathway in the mechanistic interactions leading to generation/perpetuation of AF. This evidence is further strengthened by the observation that the decreased level of phospho-MYPT-1 upon treatment with fasudil, a ROCK inhibitor, produced a reduction of Cx40 protein expression. This observation suggests that Cx40 may be a downstream target of the RhoA/ROCK pathway activation. Additional support for this hypothesis might be found via the ERK pathway control of Cxs remodeling [32,33] as ERK is one of the downstream pathways of MAPK, which is regulated upstream by ROCK [32,33].

Our extrapolation of the data obtained in circulating mononuclear cells as reflecting mechanisms taking place in myocardial cells merits a consideration. Circulating blood cells are widely used in vascular biology to study ex vivo pathophysiological mechanisms of hypertension and remodeling [34]. In addition, the role of various inflammatory mechanisms such as mononuclear leukocyte infiltration for the development of hypertensive target organ damage has been increasingly recognized in the last few years [34], and a correlation between leukocyte (polymorphonuclear and mononuclear cells), intracellular oxidative stress and hypertension has been demonstrated [35]. Leukocyte ROCK has been shown to increase leukocyte infiltration into the vascular wall resulting in the release of proinflammatory cytokines [36], and an increase in ROCK activity in peripheral leukocytes has been shown in patients with hypertension [37,38]. Finally, in an animal model of hypertension, Ocaranza and coworkers, examined the degree of association between ROCK pathway activation in circulating leukocytes with cardiac and aortic ROCK pathway activation and found that ROCK pathway activation in circulating leukocytes did reflect that in the myocardium and in the aortic wall and were significantly related to myocardial remodeling [39].

## 5. Conclusions

The data of this study point toward a role of ROCK pathway, its downstream signal MYPT-1 phosphorylation, and Cx40 in the mechanism(s) leading to AF in patients with ESRD in dialysis who represent a specific subset of patients at high risk for cardiovascular pathology and events. An exploration of the ROCK pathway in AF could contribute to provide mechanistic explanations of its involvement and further insights into AF generation in these patients and likely identify novel potential pharmacologic targets for translation into AF treatment in general.
